# A comprehensive musculoskeletal and peripheral nervous system assessment of war-related bilateral upper extremity amputees

**DOI:** 10.1186/s40779-016-0102-5

**Published:** 2016-11-15

**Authors:** Mostafa Allami, Batool Mousavi, Mehdi Masoumi, Ehsan Modirian, Hadi Shojaei, Fatemeh Mirsalimi, Maryam Hosseini, Pirouz Pirouzi

**Affiliations:** 1 Janbazan Medical and Engineering Research Center (JMERC), Tehran, Iran; 2 Emergency Medicine, School of Medicine, Qazvin University of Medical Sciences, Qazvin, Iran; 3 Candidate of Health Education and Promotion, Department of Health Education and Promotion, Faculty of Medical Sciences, Tarbiat Modares University, Tehran, Iran; 4 Department of Radiology, Imam Hospital, Tehran University of Medical Sciences, Tehran, Iran

**Keywords:** Bilateral upper extremity amputation, Musculoskeletal disorder, Peripheral nerve injury, Pain, Iraq-Iran war

## Abstract

**Background:**

Upper limb amputations are one of the unpleasant war injuries that armed forces are exposed to frequently.

The present study aimed to assess the musculoskeletal and peripheral nervous systems in Iraq-Iran war veterans with bilateral upper extremity amputation.

**Methods:**

The study consisted of taking a history and clinical examinations including demographic data, presence and location of pain, level of amputation, passive and active ranges of movement of the joints across the upper and lower extremities and spine, manual palpation, neurological examination, blood circulation pulses and issues related to a prosthetic limb. In this study, 103 Iranian bilateral upper extremity amputees (206 amputations) from the Iran-Iraq war were evaluated, and a detailed questionnaire was also administered.

**Results:**

The most common level of amputation was the finger or wrist level (108, 52.4 %). Based on clinical examination, we found high frequencies of limited active and passive joint range of movement across the scapula, shoulder, elbow, wrist and metacarpophalangeal, interphalangeal and thumb joints. Based on muscle strength testing, we found varying degrees of weakness across the upper limbs. Musculoskeletal disorders included epicondylitis (65, 31.6 %), rotator cuff injury (24, 11.7 %), bicipital tendonitis (69, 33.5 %), shoulder drop (42, 20.4 %) and muscle atrophy (19, 9.2 %). Peripheral nerve disorders included carpal tunnel syndrome in 13 (6.3 %) and unilateral brachial plexus injury in 1 (1 %). Fifty-three (51.5 %) were diagnosed with facet joint syndrome at the level of the cervical spine (the most frequent site). Using a prosthesis was reported by 65 (63.1 %), both left and right sides. The back was the most common site of pain (71.8 %).

**Conclusion:**

The high prevalence of neuro-musculoskeletal disorders among bilateral upper extremity amputees indicates that they need regular rehabilitation care.

## Background

For hundreds of years, wars have caused trauma to the limbs of soldiers, many of which result in amputation. The 8-year Iran-Iraq war was one of the longest military conflicts in the 20th century, which caused more than 1 million deaths and injuries including amputations [1]. Upper extremity amputation is one of these disastrous consequences, forcing many people to adapt to such injuries during their life [2]. Although surgical procedures on injured vessels, bones, nerves and other soft tissues has diminished the rate of amputation due to these injuries, amputation due to extremity trauma is still a main cause of morbidity in developing countries [3]. The cause of injury, socio-demographic characteristics of victims, treatment methods, infection rate and traumatic limb amputations occurring in battlefields are different from those in civilians. As war-related amputations happen mostly in young healthy persons, these unfortunate events can adversely affect the functional and physical health of the injured person, in addition to the mental and psychological sequelae that are associated with limb loss [4].

There are a few studies on the functional outcomes of amputees with upper limb amputation. These studies usually report the rates of employment, prosthesis use and demographic characteristics [5–9]. Literature on the assessment of the musculoskeletal system in “bilateral upper extremity amputation” is limited. Additionally, the generalization and comparability of the current data are often restricted by small sample sizes, different inclusion criteria and varying measures of consequences [10, 11].

There is also limited information about routine clinical examination of upper limb amputations. Successful prosthetic rehabilitation requires adequate muscle strength and normal joint motion [12, 13]. Amputees with bilateral upper extremity amputation may need to accommodate for lost wrist, forearm and/or elbow motion by extreme motion at other articulations such as teeth, chin, and lower limbs to compensate for the loss of their hand/wrist function. The risk of musculoskeletal pain may increase because of these dysfunctional movements. Musculoskeletal pain and disorders often occur in upper limb amputations [14]. Pain and other grievances may affect physical function as well [15].

Compared to the war-related lower extremity amputations, war-related upper limb amputations are much more uncommon, and its reported rate varies from 12.5 to 18.5 % across studies [1, 6–10]. Thus, there are few reports on long-term outcomes of veterans with upper limb amputation, particularly those with bilateral upper extremity amputation. As a result, some aspects of late outcomes of bilateral upper extremity amputation have not yet been well defined. This study aimed to describe clinically active and passive ranges of motion of upper extremity joints and muscle strength and to evaluate peripheral nerve and musculoskeletal disorders, distal upper extremity circulation, prosthesis use and pain of war-related bilateral upper limb amputees after more than 20 years since trauma.

## Methods

This study was conducted to assess musculoskeletal and peripheral nervous system assessments of Iranian war-related veterans with bilateral upper extremity amputation. For this purpose, a total of 140 veterans from across Iran were invited, and 103 (response rate, 73.6 %) participated. All participants were interviewed during a month. The data of the bilateral upper limb amputees was provided by the Veterans and Martyr Affair Foundation (VMAF) (VMAF offers health care services to the survivors of the 8-year Iraq-Iran war).

Each subject was visited by a fixed physical medicine and rehabilitation specialist for 2 h. In all subjects, active and passive ranges of movement of joints (if applicable), neurological status including strength testing, sensory status, deep tendon reflexes, arterial pulses, and tenderness to palpation were assessed across the upper extremities. Active and passive joint range of motion included: elevation, depression, protraction and retraction across the scapula; flexion, extension, abduction, adduction, horizontal abduction, horizontal adduction, external rotation, internal rotation across the shoulder; flexion, extension, pronation and supination across the elbow; ulnar deviation, radial deviation, flexion and extension across the wrist; flexion, extension and abduction across the metacarpophalangeal joints; flexion across the interphalangeal joints; flexion, palmar abduction, radial abduction and opposition across the thumb. Joint range of movement was graded as “normal”, “limited” and “not applicable” (due to the level of the amputation). Normal range of joint movement was determined according to the study by Gunal et al. [16]. Muscle strength was assessed using manual muscle testing and was categorized as “normal”, “good”, “fair/poor”, “trace/without movement” [17]. On sensory examination, perception to light touch and pinprick were tested. Musculoskeletal disorders included bursitis, tendonitis, and epicondylitis, which were detected on palpation and used specific clinical tests. Shoulder rotator cuff injury was detected based on shoulder specific tests. Tinel’s sign across the elbow and wrist, and phalen’s test were performed (if applicable). Cervical and lumbar range of movement tests were performed as well as palpation on the spine midline and on the facet joints. Shoulder drop and muscle atrophy were diagnosed by inspection. Deep tendon reflexes were assessed in the upper extremities, if applicable, using a hammer reflex tool. Radial and ulnar pulses were obtained via palpation and compared bilaterally, if applicable. Types of prosthetics use were also surveyed. Presence and location of musculoskeletal pain, phantom sensation and phantom pain were also asked. The subjects were asked about all the above mentioned problems simultaneously. The data for each patient were collected and entered into SPSS for Windows 22.0 (SPSS Inc., Chicago, IL., USA) for further analysis. The study was approved by the ethical committee of Janbazan Medical and Engineering Research Center (JMERC), Tehran, Iran.

## Results

Almost all the subjects were male (102, 99.0 %). Of 206 upper limb amputations, 52.4 % (*n* = 108) were at the finger or wrist, 39.8 % (*n* = 82) at the elbow and 7.8 % (*n* = 16) at the transhumeral or higher level. The mean age at the time of injury was 20.8 ± 7.6 years, and the mean age at follow-up was 37.5 ± 10.0 years. Duration of injury was approximately 16.9 ± 5.8 years. More than half of the participants (*n* = 61, 59.2 %) reported an education of 12th grade or higher, and 88.3 % (*n* = 91) of them were married. Details of the additional war-related injuries, obtained from their records, are listed in Table 1.

**Table 1:** Additional war-related injuries in bilateral upper extremity amputees (*n* = 103)

Type of injury	Left	Right	Bilateral	Total (%)
Without injuries	−	−	−	3 (2.9)
Head and Neck	−	−	−	43 (41.7)
Eyes	11	8	50	69 (67.0)
Ears	8	7	41	56 (54.4)
Lower limb	25	22	11	58 (56.3)
Trunk of the body	−	−	−	53 (51.5)
Spinal cord	−	−	−	30 (29.1)
Chemical exposure	−	−	−	6 (5.8)

**Fig. 1:**
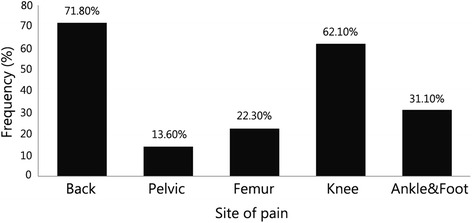
Distribution of pain in bilateral upper limb amputees

Sixty-five (63.1 %) cases reported using a prosthesis, and 20 (30.8 %) of them used their devices for ≥8 h per day. The following types of prosthesis were employed by our subjects: myoelectric (41, 39.8 %), cosmetic (22, 21.4 %), body powered (13, 12.6 %) and hybrid (3, 2.9 %). The back was the most common site of pain (71.8 %), followed by the lower limbs (Fig. 1). Phantom sensation was reported by 91 (88.3 %), and phantom pain was experienced by 61 (59.2 %) participants.

Limitations in passive ranges of motion including the scapula (*n* = 10, 1.2 %), shoulder (*n* = 23, 1.5 %), elbow (*n* = 4, 0.6 %), wrist (*n* = 59, 38.3 %), metacarpophalangeal joint (*n* = 39, 52.7 %), interphalangeal joint (*n* = 23, 47.9 %) and thumb (*n* = 51, 51.5 %) in bilateral upper limb amputees are shown in Table 2. Additionally, limitations in active ranges of motion in the scapula (*n* = 32, 7.9 %), shoulder (*n* = 118, 0.08 %), elbow (*n* = 13, 1.8 %), wrist (*n* = 73, 48.3 %), metacarpophalangeal joint (*n* = 46, 63.0 %), interphalangeal joint (*n* = 29, 60.4 %) and thumb (*n* = 63, 60.6 %) in bilateral upper limb amputees are detailed in Table 3. The most limitations in passive joint movement were found in the thumb, interphalangeal and metacarpophalangeal joints (Table 2). Additionally, the most limitation in active range of motion was observed in the thumb, interphalangeal, metacarpophalangeal and wrist joints.

**Table 2:** Passive range of motion of upper extremity joints in bilateral upper limb amputees (*n* = 103)

Joint	Right side			Left side			Unilateral/Bilateral limited	
	Limited	Normal	NA^a^	Limited	Normal	NA	*n*	%^b^
Scapula								
Elevation	0	100	3	0	101	2	0	0
Depression	0	100	3	1	100	2	0	0
Protraction	1	99	3	2	99	2	1	1.0
Retraction	2	98	3	4	97	2	2	2.0
Shoulder								
Flexion	0	96	7	1	97	5	0	0
Extension	1	95	7	1	96	6	0	0
Abduction	4	92	7	3	94	6	3	3.0
Adduction	0	96	7	0	97	6	0	0
Horizontal abduction	1	95	7	2	95	6	1	1.0
Horizontal adduction	0	94	9	2	95	6	0	0
External rotation	2	94	7	2	95	6	1	1.0
Internal rotation	2	92	9	2	95	6	1	1.0
Elbow								
Flexion	0	88	15	0	89	14	0	0
Extension	0	88	15	0	89	14	0	0
Pronation	1	85	17	1	85	17	0	0
Supination	1	84	18	1	84	18	0	0
Wrist								
Ulnar deviation	8	12	83	8	10	85	3	10.7
Radial deviation	8	13	82	7	11	85	3	10.3
Flexion	7	14	82	7	11	85	3	10.3
Extension	7	13	83	7	11	85	3	10.7
Metacarpophalangeal								
Flexion	6	7	90	7	6	90	2	10.0
Extension	6	7	90	6	6	91	2	10.5
Abduction	8	4	91	6	5	92	2	13.3
Interphalangeal								
Flexion of proximal interphalangeal	6	7	90	5	6	92	2	11.1
Extension of distal interphalangeal	6	7	90	6	5	92	2	11.1
Thumb								
Metacarpophalangeal flexion	5	7	91	5	5	93	1	5.9
Interphalangeal flexion	5	6	92	5	5	93	1	6.2
Palmar abduction	5	5	93	6	4	93	1	6.7
Radial abduction	6	4	93	6	3	94	1	6.7
Opposition	5	4	94	4	4	95	1	7.1

^a^Not applicable; ^b^The percent was calculated according to the number of applicable cases

**Table 3:** Active range of motion of upper extremity joints in bilateral upper limb amputees (*n* = 103)

Joint	Right side			Left side			Unilateral/Bilateral limited	
	Limited	Normal	NA^a^	Limited	Normal	NA	*n*	%^b^
Scapula								
Elevation	1	100	2	0	101	2	0	0
Depression	2	99	2	1	100	2	1	1.0
Protraction	3	97	3	6	95	2	2	2.0
Retraction	9	92	2	10	91	2	2	2.2
Shoulder								
Flexion	4	92	7	4	94	5	2	2.0
Extension	4	92	7	5	92	6	2	2.0
Abduction	15	82	6	13	84	6	8	8.0
Adduction	3	94	6	3	94	6	1	1.0
Horizontal abduction	10	86	7	9	88	6	5	5.0
Horizontal adduction	2	95	6	3	94	6	1	1.0
External rotation	12	84	6	9	88	6	6	6.0
Internal rotation	13	84	7	9	88	6	6	6.0
Elbow								
Flexion	1	88	14	1	88	14	0	0
Extension	1	88	14	2	87	14	0	0
Pronation	2	85	16	2	84	17	0	0
Supination	2	84	17	2	83	18	0	0
Wrist								
Ulnar deviation	11	10	82	9	9	85	4	14.3
Radial deviation	10	10	83	9	9	85	4	13.8
Flexion	7	12	84	9	9	85	4	13.8
Extension	9	10	84	9	9	85	4	14.3
Metacarpophalangeal								
Flexion	7	6	90	8	5	90	2	10.0
Extension	7	6	90	8	4	91	2	10.5
Abduction	9	3	91	7	3	93	2	13.3
Interphalangeal								
Flexion of proximal interphalangeal	7	6	90	7	4	92	2	11.1
Extension of distal interphalangeal	7	6	90	8	3	92	2	11.1
Thumb								
Metacarpophalangeal flexion	8	5	90	5	5	93	1	5.9
Interphalangeal flexion	8	4	91	5	5	93	1	6.2
Palmar abduction	7	4	92	6	4	93	1	6.7
Radial abduction	7	4	92	6	3	94	2	13.3
Opposition	6	4	93	5	3	95	2	14.3

^a^Not applicable; ^b^The percent was calculated according to the number of applicable cases

Limited muscle strength was found as follows: scapula (*n* = 95, 12.0 %), shoulder (*n* = 219, 14.6 %), elbow joints (*n* = 68, 9.9 %), wrist (*n* = 63, 52.5 %), metacarpophalangeal joint (*n* = 34, 61.8 %), interphalangeal joint (*n* = 22, 61.1 %) and thumb (*n* = 52, 68.4 %) (Table 4).

**Table 4:** Upper extremity muscle strength in bilateral upper limb amputees (*n* = 206)

Joint	Right side				Left side				Total limited	
	Normal	Good	Fair/Poor	Trace/None	Normal	Good	Fair/Poor	Trace/None	*n*	%^a^
Scapula										
Elevation	93	7	0	1	87	10	0	0	18	9.1
Depression	92	8	0	1	86	11	0	0	20	10.1
Protraction	89	8	0	3	83	7	5	2	25	12.6
Retraction	86	11	1	3	79	9	6	2	32	16.1
Shoulder										
Flexion	86	9	0	1	83	8	2	0	20	10.8
Extension	85	10	0	1	82	8	2	0	21	11.3
Abduction	77	15	2	1	77	11	4	0	33	17.7
Adduction	84	9	1	1	79	9	3	0	24	12.4
Horizontal abduction	82	11	2	1	78	9	4	0	27	14.5
Horizontal adduction	83	11	0	1	78	9	3	0	24	12.9
External rotation	75	16	3	1	77	11	3	1	35	18.8
Internal rotation	74	16	3	1	77	11	3	1	35	18.8
Elbow										
Flexion	78	6	1	2	75	8	0	1	18	10.5
Extension	78	6	1	2	75	8	0	1	18	10.5
Pronation	76	5	1	1	71	9	0	1	17	10.4
Supination	76	4	1	1	71	8	0	1	15	9.3
Wrist										
Ulnar deviation	7	3	1	5	7	2	0	5	16	53.3
Radial deviation	7	3	1	5	7	2	0	5	16	53.3
Flexion	7	5	0	4	7	2	0	5	16	53.3
Extension	8	4	1	3	7	2	0	5	15	50.0
Metacarpophalangeal										
Flexion	4	1	2	4	3	0	2	4	13	65.0
Extension	4	1	2	4	3	0	2	3	12	63.2
Abduction	4	0	3	3	3	0	1	2	9	56.3
Interphalangeal										
Flexion of proximal interphalangeal	4	0	3	4	3	0	2	2	11	61.1
Extension of distal interphalangeal	4	0	4	3	3	0	2	2	11	61.1
Thumb										
Metacarpophalangeal flexion	3	1	4	2	2	0	1	3	11	68.8
Interphalangeal flexion	2	1	4	2	2	0	1	3	11	73.3
Palmar abduction	2	1	3	2	3	0	1	3	10	66.7
Radial abduction	2	1	3	2	3	0	1	3	10	66.7
Opposition	2	0	3	3	3	0	1	3	10	66.7

^a^The percent was calculated according to the number of applicable cases

The most common finding among all musculoskeletal problems was bicipital tendonitis (*n* = 69, 33.5 %). Epicondylitis was also frequently discovered (65, 31.6 %). Rotator cuff injury was another issue found in 24 participants (11.7 %), with the same frequency in the right and the left sides. Shoulder bursitis was observed in 2 participants (1.0 %) without elbow bursitis in any subject. Additionally, shoulder drop was found in 42 (20.4 %) subjects. Muscle atrophy was less common compared with the other problems (*n* = 19, 9.2 %).

Fifty-three (51.5 %) cases had facet joint syndrome at the level of the cervical spine; 13 (12.6 %) cases at the level of the lumbar spine and only 1 (1 %) case at the level of the thoracic spine. Regarding peripheral nerve injuries, carpal tunnel syndrome symptoms were found in 13 participants (6.3 %), and unilateral brachial plexus injury was only observed in 1 (1 %) case. Weak distal upper extremity circulation was only observed in 1 (1.0 %) subject.

## Discussion

After more than 20 years of disability, war-related bilateral upper limb amputees were all visited by a PM&R specialist. Our findings revealed that a considerable amount of these subjects suffered from musculoskeletal disorders and pain. Most of the subjects were young at the time of casualty and had additional injuries, especially in their lower limbs.

More distal amputation was associated with a higher rate of small joint limitation. Muscle strength in the shoulder and elbow joints was consistent with previous results, which may be due to having the same measurements. Most cases did not have any movement problems in their other joints, and limited joint motion in other parts of the body was observed in the lumbar region, ankle, and foot which was in contrast with a study that reported that mobility of the neck joint was reduced bilaterally [18].

Approximately two-thirds of the survivors suffered from pain in various parts of their bodies, especially back pain, which was followed by knee pain. Consistent with our results, back pain in upper limb amputees was reported as the most common complaint in up to more than half of the participants [15]. Moreover, consistent with previous results, more than three-quarters of our cases experienced phantom sensation [7]. Phantom pain was found in approximately two-thirds of participants, which was in accordance with the estimates of previous studies of 50 to 90 % in upper limb amputees [15, 19–21]. As pain control is important for patient comfort and health, prevention and treatment of pain can play a significant role in upper limb amputation rehabilitation.

The greatest limitation in passive and active joint movement was found in the thumb and interphalangeal and metacarpophalangeal joints. Similarly, metacarpophalangeal, interphalangeal and thumb joints demonstrated the weakest muscle strength, which emphasized the importance of exercises to use these muscles. The results of the unilateral upper limb amputees’ survey indicated active joint motion, especially in shoulder flexion, was limited, whereas forearm rotation and muscle strength were less limited [18]. Regarding range of motion of joints, scapula retraction, shoulder abduction, elbow pronation and supination and wrist ulnar and radial deviation were the dominant limited movements due to muscle contracture.

According to the musculoskeletal and peripheral nerve examinations, bicipital tendonitis was the most prevalent complication found in approximately one-third of the subjects. Lateral epicondylitis was the next most frequent complication observed. Development of bicipital tendonitis and lateral epicondylitis indicated elbow joint overuse in subjects with elbow amputation. The elbow is susceptible to repeated stresses, and the development of bicipital tendonitis and lateral epicondylitis due to overuse can occur [22].

There is limited research on overuse syndrome in bilateral upper limb amputation, which is associated with rotator cuff injuries including tendonitis and tears, shoulder impingement, and bursitis [14, 15, 23]. Additionally, rotator cuff syndrome and lateral epicondylitis on the non-amputated side were more common than in previous studies [14]. As the frequency of below elbow amputation was higher than above elbow amputation, a higher prevalence of epicondylitis was predictable. In a report of peripheral nerve problems in bilateral upper limb amputation, carpal tunnel syndrome was estimated to occur in more than one-fourth of proximal upper limb amputees [24]. The occurrence of carpal tunnel syndrome in the present study was estimated to be more than one-third, which is higher than that in previous studies on bilateral and unilateral upper limb amputees [14, 24]. Moreover, musculoskeletal disorders were more prominent than peripheral nerve injuries among bilateral upper limb amputees. We found no previous report in our literature review regarding facet joint syndrome in the cervical spine using symptoms and signs including neck pain, local spine tenderness and decreased range of motion, which was the next most common musculoskeletal disorder in our subjects.

Global prosthesis use has been estimated to be up to 80 %, and two-thirds of participants in a previous study employed the prosthesis for ≥8 h/day [12, 15, 21]. In contrast, prosthesis use was less frequent in the participants in this study, and only one-quarter employed prosthesis for ≥8 h/day. The use of myoelectric versus cosmetic prostheses can increase actual prosthesis use in activities of daily living (ADL) [21]. In contrast, rejection of prosthesis use was reported because of poor cosmetic quality of some types of devices. Additionally, unilateral upper limb amputees prefer cosmetic hands, whereas among bilateral upper limb amputees, hook hands and functional hands were the most frequently used types of prosthetic limbs [15]. Unlike previous results, the most prevalent types of prosthetic limbs currently used by our participants included myoelectric and cosmetic hands.

### Limitations

The time limit was a substantial issue in obtaining a comprehensive pain investigation including pain inventory scale and visual analog scale. We also did not have a psychologist to perform physical body assessments. We had no access to EMG/NCS, ultrasound or X-rays at the time of the assessments. We also had no access to the records of their past medical visits or rehabilitation services to compare with their current conditions.

### Recommendation

Further studies of subjects who had brachial plexopathy/peripheral neuropathy should investigate the reasons for non-use of particular types of prostheses, and training on the use of the prosthesis seems necessary.

## Conclusions

This study found varying frequencies of limited active and passive ranges of movement and muscle strength among bilateral upper limb amputees. Our findings have shown that bilateral upper limb amputees are at high risk of neuromusculoskeletal disorders, including lower back pain, lower extremity pain, cervical pain, soft tissue problems such as tendinitis and bursitis in the shoulder, phantom limb pain and sensation, and carpal tunnel syndrome. This research will help primary health providers identify upper limb amputees with high rehabilitation needs. We recommend more studies to investigate clinical examinations and the effect of prosthesis wear on the musculoskeletal system in bilateral upper limb amputees. Encouragement to do regular appropriate exercises and routine visits by specialists seems to be necessary in this population. Moreover, counseling these subjects about the risks of musculoskeletal disorders seems to be essential. Additionally, encouragement of family members to maintain their emotional support and have good relationships with them would be helpful. We also recommend more studies on pain syndromes in bilateral upper limb amputees and psychological aspects of pain.

## Abbreviations

ADL: activity of daily living
EMG/NCS: electromyography/nerve conduction study
JMERC: Janbazan medical and engineering research center
NA: not applicable
PM&R: physical medicine and rehabilitation
SPSS: statistical package for the social sciences
VMAF: veterans and martyr affair foundation
